# Exploring Severe Mental Illness and Diabetes: Protocol for a Longitudinal, Observational, and Qualitative Mixed Methods Study

**DOI:** 10.2196/13407

**Published:** 2019-09-06

**Authors:** Sue Bellass, Johanna Taylor, Lu Han, Stephanie L Prady, David Shiers, Rowena Jacobs, Richard Ian Gregory Holt, John Radford, Simon Gilbody, Catherine Hewitt, Tim Doran, Sarah L Alderson, Najma Siddiqi

**Affiliations:** 1 Mental Health and Addiction Research Group Department of Health Sciences University of York York United Kingdom; 2 Department of Health Sciences University of York York United Kingdom; 3 Psychosis Research Unit Prestwich Hospital Greater Manchester Mental Health NHS Foundation Trust & The University of Manchester Manchester United Kingdom; 4 University of Keele Keele United Kingdom; 5 Centre for Health Economics Department of Health Sciences University of York York United Kingdom; 6 Faculty of Medicine University of Southampton Southampton United Kingdom; 7 DIAMONDS VOICE Patient and Public Involvement Panel, Bradford District Care NHS Foundation Trust Bradford Bradford United Kingdom; 8 York Trials Unit Department of Health Sciences University of York York United Kingdom; 9 Faculty of Medicine and Health University of Leeds Leeds United Kingdom

**Keywords:** schizophrenia, bipolar disorder, diabetes mellitus, diabetes complications

## Abstract

**Background:**

The average life expectancy for people with a severe mental illness (SMI) such as schizophrenia or bipolar disorder is 15 to 20 years less than that for the population as a whole. Diabetes contributes significantly to this inequality, being 2 to 3 times more prevalent in people with SMI. Various risk factors have been implicated, including side effects of antipsychotic medication and unhealthy lifestyles, which often occur in the context of socioeconomic disadvantage and health care inequality. However, little is known about how these factors may interact to influence the risk of developing diabetes and poor diabetic outcomes, or how the organization and provision of health care may contribute.

**Objective:**

This study aims to identify the determinants of diabetes and to explore variation in diabetes outcomes for people with SMI.

**Methods:**

This study will employ a concurrent mixed methods design combining the interrogation of electronic primary care health records from the Clinical Practice Research Datalink (CPRD GOLD) with qualitative interviews with adults with SMI and diabetes, their relatives and friends, and health care staff. The study has been funded for 2 years, from September 2017 to September 2019, and data collection has recently ended.

**Results:**

CPRD and linked health data will be used to explore the association of sociodemographics, illness, and health care–related factors with both the development and outcomes of type 2 diabetes in people with SMI. Experiences of managing the comorbidity and accessing health care will be explored through qualitative interviews using topic guides informed by evidence synthesis and expert consultation. Findings from both datasets will be merged to develop a more comprehensive understanding of diabetes risks, interventions, and outcomes for people with SMI. Findings will be translated into recommendations for interventions and services using co-design workshops.

**Conclusions:**

Improving diabetes outcomes for people with SMI is a high-priority area nationally and globally. Understanding how risk factors combine to generate high prevalence of diabetes and poor diabetic outcomes for this population is a necessary first step in developing health care interventions to improve outcomes for people with diabetes and SMI.

**Trial Registration:**

ClinicalTrials.gov NCT03534921; https://clinicaltrials.gov/ct2/show/NCT03534921

## Introduction

### Background

Severe mental illness (SMI) refers to a set of disabling conditions such as schizophrenia, schizoaffective disorder, or bipolar disorder. People with SMI, who account for around 1% of the population [1], may experience feelings of persecution, hallucinations, problems with mood, impaired cognition, and lack of motivation. These difficulties can have an adverse impact on several areas of life such as housing, employment, relationships, and personal care, which can, in turn, increase the likelihood of mental and physical health problems. They have a reduced life expectancy, living for 15 to 20 years less than the population as a whole, and experience poorer health outcomes [2-5].

Diabetes is a significant contributor to the increased morbidity and mortality experienced by people with SMI. The condition is 2 to 3 times more prevalent in this population [6-8], and complications of diabetes are higher than for people without SMI [9,10]. The link between the metabolic side-effects of antipsychotic medication and diabetes has been well-documented [11-13], but there are additional complexities; other work points to the possibility of a genetic neuroinflammatory mechanism predisposing people toward both conditions [14], which could explain the occurrence of the comorbidity independent of antipsychotic medication [15,16]. Furthermore, other factors have been implicated such as lifestyle health risk factors such as diet [17], smoking [18,19], low levels of physical activity [20], and higher levels of other comorbid conditions [21]. However, little is known about the relative contribution of these factors, or about possible synergistic relationships that may increase the risk of people with SMI developing diabetes or experiencing poorer diabetes outcomes.

There is strong evidence that people with SMI are more likely to be socially disadvantaged than people without SMI [22-24], experiencing reduced access to material, financial, social, or structural resources such as transport, childcare, paid leave, and advocacy services [25]. Accordingly, people with SMI have difficulty navigating health care systems, less capacity to take advantage of health promotion opportunities, and encounter more barriers to taking up interventions designed to prevent or treat illness. Moreover, despite calls for integrated service models that treat the *whole person* [26], such models of service delivery can be thwarted by rigid boundaries between primary and secondary care, unclear practitioner accountability for mental and physical health care [27,28], and diagnostic overshadowing (misattributing physical health problems to mental illness) [29].

There is conflicting evidence about the quality of diabetes care for those with and without SMI [10,30-34]. In addition, little is known about the costs of diabetes screening, monitoring, and management for this group, or about the relationship between diabetes interventions and health outcomes. Furthermore, although a recent study demonstrated that diabetes distress—the emotional burden of managing a serious, chronic condition—is known to significantly affect people with diabetes who do not have SMI [35], there is limited evidence on how diabetes might impact upon the mental health of people who already experience psychological vulnerabilities.

Improving diabetes care for people with SMI is a high priority nationally and globally [36,37]. Understanding how SMI and other risk factors combine to generate high diabetes prevalence and poor diabetes outcomes and how the quality and quantity of health care services and interventions can impact on these risk factors is a necessary first step in developing health care interventions to improve outcomes for people with diabetes and SMI.

### Objectives

This study aims to identify the determinants of diabetes in people with SMI and to explore variation in diabetes outcomes for people with SMI to develop potential health care interventions that can be tested further.

The study has the following objectives:

1. In people with SMI, to identify which sociodemographic, illness, family history, and lifestyle factors are associated with the development of diabetes
2. In people with SMI and diabetes, to identify which sociodemographic, illness, family history, and lifestyle factors are associated with variation in diabetes and mental health outcomes
3. In people with SMI, to compare health care interventions, physical and mental health outcomes in those with diabetes, and those without diabetes
4. In people with diabetes, to compare health care interventions, physical and mental health outcomes in those with SMI, and those without SMI
5. To understand the factors that influence access to, and receipt of, diabetes care for people with SMI and explore the experience of diabetes health care by people with SMI
6. To compare diabetes care provision for people with and without SMI, and estimate costs for these
7. To identify which health care interventions (eg, medication, referrals, and care pathways) may be associated with better diabetes outcomes for people with SMI and diabetes.

## Methods

### Design

The underpinning theoretical framework for the study conceptualizes socioeconomic conditions as a fundamental cause of health inequalities [25]. Under a social inequalities framework, a concurrent triangulation mixed methods design will be used comprising (1) a quantitative longitudinal observational study of anonymized individual patient records of adults diagnosed with SMI in Clinical Practice Research Datalink (CPRD) and (2) a qualitative interview study of people with coexisting SMI and diabetes, their family or friends, and health care staff involved in diabetes and mental health care. Both workstreams will be informed by a synthesis of existing evidence.

**Figure 1:**
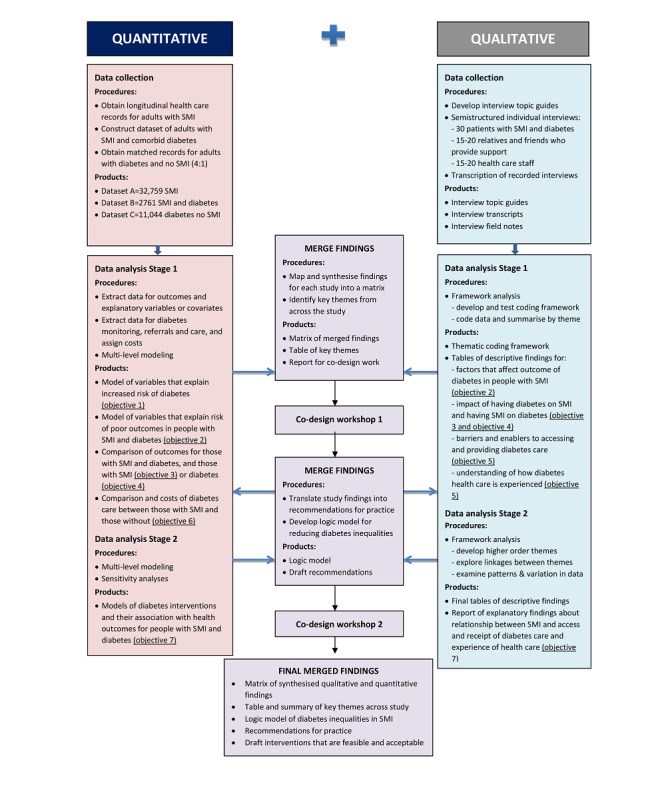
Study Flow Diagram.

The mixed methods design is underpinned by a pragmatic paradigm, which acknowledges that data types, when integrated together, will enable the development of a more complete understanding of health inequalities in this population than would be possible from either method alone [38]. Interrogation of quantitative data from CPRD will be used to generate insights into inequalities through identifying trends, patterns, and correlations, indicating which groups are more at risk of developing diabetes or experiencing poor diabetes outcomes. These data will be complemented by findings from the analysis of qualitative interviews conducted to explore the difficulties people with SMI have in managing their diabetes and accessing services. The quantitative and qualitative data will be integrated iteratively as the study progresses; key findings emerging from the CPRD analysis will be explored further in interviews, and qualitative themes will be used to inform the choice of variables that can be explored in the quantitative dataset (see Figure 1). Key to both the quantitative and qualitative analyses will be exploring the effects of disadvantage and deprivation on risks, outcomes, and experiences for people with SMI and diabetes.

### Patient and Public Involvement

The study is supported by DIAMONDS VOICE, a patient and public involvement (PPI) panel that contributes to a wider research program called Diabetes and Mental Illness: Improving Outcomes and Services. The panel was involved in prioritizing the research questions during the design phase of this study and reviewed all patient and public-facing documentation before submission to the National Health Service Research Ethics Committee. The panel will continue to contribute to the study in the following ways: advising on how to ask sensitive questions, acting as practice interviewees, advising on minimizing participant burden, promoting the study at local events, and advising on recruitment and dissemination strategies. Both the project management team and the study steering committee have service user representation to ensure that the patient perspective is incorporated in project decisions.

### Ethical Approval and Data Use Agreement

A data use agreement for CPRD records and linked Hospital Episode Statistics (HES) and Office for National Statistics (ONS) mortality data was granted by the International Scientific Advisory Committee (ref: 17_161R). Approvals for the qualitative study were granted by the National Institute of Health Research Health Research Authority and the Greater Manchester West Research Ethics Committee (ref: 18/NW/0005), following a submission made through the Integrated Research Application System (ref: 235328).

The study is registered on the NIHR Central Portfolio Management System (CPMS; ref: #37024) and ClinicalTrials.gov (record identifier #NCT03534921).

### Quantitative, Longitudinal, and Observational Study

This workstream examines the impact of potential risk factors on the development and the time to onset of diabetes in people with SMI and variation in mental and physical health outcomes in people with comorbid SMI and diabetes. Diabetes health care and outcomes for people with SMI and diabetes will be compared with outcomes for people with either condition alone. Variations in diabetes screening, monitoring, and management will be examined and costs estimated. The role of these interventions in contributing to health outcomes will be explored.

#### Study Population

The primary study population comprises adult patients (age ≥18 years) living in England who are registered with a general practice contributing up to research standard data to CPRD and who have remained within CPRD for the study period, from April 1, 2000, to March 31, 2016.

#### Datasets

CPRD is the world’s largest computerized database of anonymized longitudinal medical records from primary care. Information includes records of symptoms, diagnoses, referrals, prescriptions issued in primary care, records of immunizations and vaccinations, laboratory test results, lifestyle information (eg smoking and alcohol status), and all other types of care administered as part of routine general practice. Currently, data are collected on over 5 million active patients from 674 general practices, covering around 6.9% of the UK population registered with a general practitioner [39].

Records for 3 population groups will be extracted from CPRD. The datasets will be structured as shown in Table 1.

#### Data Linkages

Electronically linked data for individuals in the study population will be obtained from the following sources: HES data for hospital admissions [41], ONS for dates and causes of death [42], and the Index of Multiple Deprivation (IMD) for area deprivation at practice and patient level [43].

#### Health and Health Care Outcomes

The following outcomes listed in Table 2, corresponding to the objectives described above, will be examined.

Analyses may be limited by the quality and availability of data for some of the above variables, but wherever possible, validated code lists will be employed.

**Table 1:** Structure of the Clinical Practice Research Datalink datasets.

Dataset	Population	Specification	Estimated sample size of patients
Dataset A	Adults (≥18 years) with SMI^a^	Longitudinal health care records of adult patients with SMI defined as the presence of a clinical diagnostic code for schizophrenia, affective disorder (divided into bipolar or unspecified affective disorder), and other types of psychoses. Read codes previously tested and applied by the research team [37] will be used to identify the presence of SMI.	33,000
Dataset B (a subset of dataset A)	Adults with a diagnosis of SMI and diabetes mellitus	Diabetes, identified using previously tested and validated Read codes, will be further defined as the presence of a clinical diagnostic code for type 1 diabetes, type 2 diabetes, drug-induced diabetes, or unspecified diabetes. Individuals with a diagnostic code of gestational diabetes, cystic fibrosis, and hemochromatosis will be excluded.	3600 based on a predicted diabetes prevalence of 11.1% [40]
Dataset C (matched controls to dataset B)	Adults with a diagnosis of type 2 diabetes but without SMI	Patients with type 2 diabetes in dataset B will be matched by Clinical Practice Research Datalink to a cohort of patients who have a diagnosis of type 2 diabetes but without SMI (controls) with a case to control ratio of 1:4 on the basis of age, gender, and practice.	14,400

^a^SMI: severe mental illness.

**Table 2:** Health and health care outcomes corresponding to objectives.

Outcome	Objective #
Diabetes status and onset	1
Diabetic and cardiovascular control (measured by recorded hemoglobin A_1c_, blood pressure, and cholesterol levels)	2
Diabetic complications: acute hyperglycemic events, hypoglycemia, microvascular complications (retinopathy, neuropathy, and nephropathy)	2, 4
Diabetic complications: macrovascular complications (coronary artery disease, cerebrovascular disease, and peripheral arterial disease)	2, 3, 4, 7
Hospital admissions for the above conditions	2, 3, 4, 7
Mental health outcomes including severe mental illness relapses (measured by hospital admissions and general practitioner referrals to community mental health teams or crisis teams) and markers of depression or anxiety (ie, general practitioner diagnoses or prescriptions for antidepressants)	2, 3, 4, 7
Mortality	2, 3, 4, 7
Health care utilization (including the number and type of primary care consultations) and costs	6
Health care interventions (eg, first and second generation antipsychotic and antidiabetic medications, care pathways, and referrals)	6, 7

#### Risk Factors and Explanatory Variables

Known risk factors and covariates for developing diabetes and diabetes complications will be included in the analyses to determine their relative association with the development and progress of diabetes in people with SMI. The list of factors will be determined by evidence synthesis, complemented by expert consensus, and will be responsive to emerging findings from the qualitative interviews.

The candidate risk factors include patient demographics and socioeconomic markers (sex, age, ethnic group, area deprivation, and rurality), lifestyle (obesity, smoking, and alcohol consumption), social vulnerability markers such as housing status, hyperlipidemia, family history, type of SMI and diabetes (including order and timing of diagnosis), length of diagnosis, illness severity, and multimorbidity.

#### Statistical Analysis

It is anticipated that there will be around 5 to 6 years of data on each individual, which will enable more robust analyses than cross-sectional data would permit. Longitudinal data afford better control for unobserved characteristics, at either the individual or practice level that plausibly impact both practice performance and patient outcomes (eg, practice style and culture), and which may otherwise confound important relationships. In addition, repeated observations at practice or patient level allow the investigation of lags in the relationship between diabetes management and outcomes, as the timing of events can be observed. Both these factors are crucial in identifying plausible causal mechanisms linking diabetes management to outcomes.

The potential for confounding will be addressed by matching on index date to avoid inclusion of *ghost* patients, conducting sensitivity analyses, and evaluating the potential for unmeasured confounding and the size of any observed effects.

A range of regression models will be used for statistical analyses, taking account of the hierarchical structure of the data, where, for example, activities are *nested within* patients who in turn are nested within practices. Linear, logistic and survival regressions will be applied as appropriate depending on the outcome variable of interest. Multilevel mixed effects will be estimated to account for the correlation in the longitudinal health records of the same patient, as well as the unobserved correlation at practice level. An outline of planned analyses by study objective is given in Table 3.

Under all objectives, analyses will be conducted in line with the inequalities framework to quantify the absolute and relative effect of social inequalities on quality of care and outcomes. Under objectives 1 and 3, the disparities will be modeled within the SMI population, under objective 2 within the SMI and diabetes population, and both within and between the diabetes populations (SMI and non-SMI) under objectives 4 to 7. Specifically, where sample size permits, analyses will be stratified, for example, by ethnicity and or deprivation and disadvantage markers such as IMD; housing status and rurality will be used as independent variables to estimate gap or gradient effects.

All statistical models will include a set of relevant patient, local population, and practice covariates where possible to control for confounding and interacting influences potentially masking the relationship between diabetes management and outcomes.

In sensitivity analyses, noncompliance (including refusal of treatment, informed dissent, and nonattendance) will be included as an independent variable in the models. Sensitivity analyses will be conducted with noncompliance treated as either a time-dependent (*expiring*) or time-independent (*nonexpiring*) variable and as specific to the refused treatment or as a general marker of noncompliance. Noncompliance will be used to create *offer of treatment* explanatory variables, facilitating *intention-to-treat* type analyses.

Robustness checks will be carried out to ensure our results are reliable, and plausibility tests to ensure findings are meaningful in practice and can inform policy. Model assumptions will be checked for all analyses, and if they are in doubt, the data will be transformed before analysis or alternative nonparametric analysis methods will be used.

**Table 3:** Summary of statistical analysis plan by study objective.

Objective #^a^	Description	Datasets	Variables	Analysis
1	The impact of key explanatory variables on both diabetes status and time to onset of diabetes	Quantitative dataset A (people with SMI^b^)	Explanatory variables: sociodemographic characteristics, medication use, physical and mental health status, family history of diabetes, biometric data dysregulation, and lifestyle factors	Multilevel modeling: logistic model (diabetes status) and survival model (time to diabetes onset)
2	The impact of key explanatory variables (as above) on diabetes and mental health outcomes	Quantitative dataset B (people with SMI and type 2 diabetes)	Outcomes: diabetic and cardiovascular control; diabetic complications; hospital admissions; mental health outcomes, for example relapses and episodes of depression and anxiety and mortality	Repeated measures mixed models: linear, logistic, and survival models
3	The impact of diabetes status and other explanatory variables (as above) on physical and mental health outcomes	Quantitative dataset A	Outcomes: macrovascular diabetic complications, hospital admissions, mental health outcomes, and mortality	Poisson or negative binomial multilevel models for count outcomes, logistic multilevel models for binary outcomes
4	The impact of SMI status and other explanatory variables (as above) on physical and mental health outcomes	Quantitative datasets B and C (matched cohort of non-SMI patients with diabetes)	Outcomes as above	Similar multilevel modeling to objective 3
6	Comparison of, and cost estimation for, diabetes health care provision for people with and without SMI	Quantitative datasets B and C	Contacts with primary care staff and hospitalization but not medication costs: Health care costs will be calculated by attaching unit costs to contacts recorded in the Clinical Practice Research Datalink database and also hospital inpatient episodes, from the linked Hospital Episode Statistics data. National average costs will be calculated using National Health Service Reference Costs and Personal Social Services Research Unit costs.	Cost data will be modeled on patient level as a nonlinear function (such as exponential) of covariates to take into account the nonnegative, highly skewed, and leptokurtic characteristics. We will choose the model depending on the distribution of the cost data. Random intercepts will be estimated to capture the baseline differences in health care provision at practice level.
7	Impact of SMI status and other explanatory variables (as above) on whether or not someone receives a diabetes intervention	Quantitative datasets B and C	Diabetes interventions, for example, regular reviews, monitoring, referral to education programs, foot checks, retinopathy screening, and referrals to secondary care. Outcomes, for example, diabetes admissions and diabetic complications	The probability of receiving interventions will be modeled as a function of SMI status, patient characteristics, and other key predictors. Random intercepts at practice level will be included in the model to capture the systematic differences in service provision.

^a^Objective number 5 will be explored in the qualitative workstream.

^b^SMI: severe mental illness.

### Qualitative Interview Study

Under the inequalities framework, the qualitative study aims to develop understandings of the factors that influence access to and receipt of diabetes care and how health care service provision is perceived by people living with SMI and diabetes, those who support them, and health care professionals. The interviews will be conducted in person or over the telephone, using topic guides that have been informed by expert consultation, existing literature, and preliminary CPRD analyses.

#### Population

Study participants will include (1) adults with SMI and diabetes (excluding gestational diabetes) living in the community (n=30-50), (2) relatives or friends who are involved in the care of a person with SMI and diabetes (n=15-20), and (3) health care staff (commissioners, clinicians, nurses, and other health care staff who are involved in health care services for people with SMI and diabetes; n=15-20).

#### Sampling

People with SMI and diabetes will be included in the study if they are:

- Aged 18 years or older
- Diagnosed with a SMI and not currently experiencing an acute relapse
- Have a diagnosis of diabetes (excluding gestational diabetes)
- Live in the community
- Have capacity to consent to the study.

Maximum variation purposive sampling will be used, informed by demographic and illness characteristics identified during the descriptive analysis of CPRD data and the social inequalities theoretical framework to gain understandings of the diversity of experience in this group. People with SMI and diabetes will be sampled from rural and urban areas, areas of wealth and deprivation, and areas with diverse communities.

To ensure representation from different health care disciplines, health care staff will be sampled purposively. GPs, practice nurses, diabetes nurses, mental health nurses, case managers, psychiatrists, and diabetologists will be invited to take part in the study.

The sampling strategy in all groups will be continually monitored as the study progresses to ensure that diversity of experience is captured. Recruitment will continue until data saturation is reached, that is, no new themes are emerging from the interviews [44].

#### Recruitment

Evidence suggests that around 20 to 30% of people with SMI are supported solely within primary care [45]. Therefore, to understand how people with SMI experience diabetes care, it is important to sample from both general practices and specialist mental health services. Patients and relatives will be identified by general practice and mental health service staff using database and caseload searches. Following screening by a clinician, eligible patients will be sent a study pack containing an invitation letter, a participant information sheet, a response form, and a prepaid return envelope.

Patients will also be recruited via existing research cohorts and clinic and website advertisements. Patients from existing cohorts who have agreed to be contacted for future research will receive a study pack. Patients or relatives who express an interest in participating after seeing an advertisement will also be provided with the pack. Patients who contact the study team will be asked to provide permission to access medical records to screen for eligibility.

To recruit relatives or friends to the study, participating patients will be asked to identify a person who supports them who will then be approached by the research team. Relatives who are known to clinicians will also be provided with a study pack.

For health care staff recruitment, lead clinicians in participating general practices and mental health services will be asked to identify health care staff with experience of providing services to this population.

#### Consent

Written or audio-recorded informed consent will be obtained from all participants. Study information will be provided (written and verbal) using materials that have been developed with the PPI Group. Capacity to participate will be assessed by staff with appropriate experience during contact telephone calls and again before interview.

#### Data Collection

All interviews will be conducted using semistructured topic guides (see Table 4), which will be informed by the evidence synthesis and consultation with the PPI Group and the project team. Data collection will begin in April 2018, and it is anticipated that it will be completed by the end of December 2018. The topic guide will be reshaped iteratively as the project progresses, being influenced by developing themes, CPRD analyses, and new evidence that emerges in the field.

Interviews will be conducted by an experienced qualitative researcher and will last for a maximum of 90 min (patients and relatives) or 45 min for health care staff. With participants’ permission, interviews will be audio-recorded.

**Table 4:** Qualitative interview topics by participant group.

Participant group	Topic areas
Patients with severe mental illness and diabetes	Emergence of the conditions and experience of diagnosis Day-to-day experiences of living with the comorbidities including self-management and how morbidities impact one another Experience of accessing and receiving health or other support services Suggestions for improvements to services
Relatives and friends who provide support	Experiences of providing support The impact of the comorbidity on shared activities of daily life Perceptions of support received from formal services for the person they care for and themselves Perceptions of barriers and facilitators to accessing care Suggestions for improvements to services
Health care staff	Role in supporting people living with this comorbidity Perceptions of the challenges faced by people living with the comorbidity Perceptions of barriers and facilitators to integrating physical and mental health services Perceptions of staff training needs Suggestions for improvements to services

#### Data Analysis

Data will be analyzed using NVivo 11 (QSR International). The framework method [46] will be employed, which combines deductive analysis of a priori themes identified in the evidence synthesis, the quantitative analysis, and through expert consultation, with inductive analysis of themes that emerge from the data. Analysis comprises a 5-stage process of scaffolding (identifying and extracting themes), indexing (labeling and sorting data to test the framework), coding (coding the data to the framework), descriptive analysis (categorizing and classifying data into higher order themes), and explanatory analysis (detecting thematic patterns and relationships). Each group of participants will be analyzed separately before cross-group analyses will be undertaken to triangulate the qualitative data. Areas of convergence and divergence within and across the 3 groups of data will be explored, and possible explanations for contradictory findings will be proposed. To ensure dependability and credibility [47], the key steps of the analysis will be conducted by at least two researchers, and the framework will be reviewed regularly by the project team and PPI panel.

## Results

The study was funded for 2 years, from September 2017 to September 2019. Data collection is ongoing in the qualitative workstream and is expected to be completed by the end of December 2018.

In the later stages of analysis, the findings from the qualitative study will be merged with quantitative analyses using established integration techniques such as comparison matrices to validate, confirm, or refute findings, and joint data displays [48]. These analyses will be used by the project team to create a logic model drawing on process evaluation methodology [49]. The model will delineate how diabetes contributes to health inequalities in people with SMI and diabetes, exploring the relationship between health care provision and inequalities. Analytic rigor will be assured through regular discussion of analytic strategies with the project team, study steering committee, and PPI group.

Findings will be considered in 2 multistakeholder workshops involving service users, family members, researchers, clinicians, commissioners, health service managers, and representatives from third sector organizations to develop recommendations to improve diabetes care for people with SMI. The first workshop will review the merged findings, identify further analyses that can be conducted, and will create draft recommendations to improve health care. The second workshop will use the final analyses to refine the draft recommendations and to design and assess the feasibility of interventions or care pathways where the need for these has been identified empirically.

A range of channels will be used to communicate study findings to participants, stakeholders, and likely beneficiaries of the research. Key audiences will include patients, family members, health care staff, policy makers, voluntary and commercial sector organizations, and researchers. Representatives from these sectors will be invited to a dissemination event at the end of the study.

Research outputs will take the form of written reports, journal articles, oral presentations, and content distributed through international, national, and local networks, websites, and social media.

## Discussion

People with SMI face significant health inequalities compared with the population as a whole, experiencing poorer physical health and having a reduced life expectancy of 15 to 20 years. Diabetes contributes to this inequality, occurring 2 to 3 times more often in people with SMI. Although various sociodemographic, genetic, illness, and health care–related factors are thought to increase the risk of developing the condition, the relative influence of these factors, and how they might interrelate, remains poorly understood. Furthermore, evidence suggests that people with comorbid SMI and diabetes experience poorer diabetic outcomes than those without SMI.

This research will provide greater understandings of why people with SMI are more at risk of developing diabetes and why their diabetic outcomes may be poorer than those without SMI. Associations between diabetes screening, monitoring and management, and health outcomes will be investigated to identify interventions with the potential to improve outcomes in this population. In addition, exploring the health care needs and health care delivery concerns of people with comorbid SMI and diabetes, relatives or friends who support them, and health care staff will enable the development of a more comprehensive understanding of the factors that contribute to poor outcomes and the drivers of improved health outcomes in SMI and diabetes.

## Abbreviations

CPRD: Clinical Practice Research Datalink
HES: Hospital Episode Statistics
IMD: Index of Multiple Deprivation
ONS: Office for National Statistics
PPI: patient and public involvement
SMI: severe mental illness
